# Systematic Bioinformatic Analyses of Nutrigenomic Modifications by Polyphenols Associated with Cardiometabolic Health in Humans—Evidence from Targeted Nutrigenomic Studies

**DOI:** 10.3390/nu13072326

**Published:** 2021-07-06

**Authors:** Tatjana Ruskovska, Irena Budić-Leto, Karla Fabiola Corral-Jara, Vladimir Ajdžanović, Anna Arola-Arnal, Francisca Isabel Bravo, Georgia-Eirini Deligiannidou, Jaroslav Havlik, Milkica Janeva, Elena Kistanova, Christos Kontogiorgis, Irena Krga, Marika Massaro, Marko Miler, Verica Milosevic, Christine Morand, Egeria Scoditti, Manuel Suárez, David Vauzour, Dragan Milenkovic

**Affiliations:** 1 Faculty of Medical Sciences, Goce Delcev University, 2000 Stip, North Macedonia; tatjana.ruskovska@ugd.edu.mk (T.R.); milkica.janeva@ugd.edu.mk (M.J.); 2 Institute for Adriatic Crops and Karst Reclamation, 21000 Split, Croatia; irena.budic-leto@krs.hr; 3 Unité de Nutrition Humaine (UNH), Université Clermont Auvergne, Institut National de Recherche pour L’agriculture, L’alimentation et L’environnement (INRAE), Faculté de Médecine, F-63000 Clermont-Ferrand, France; karla-fabiola.corral-jara@inrae.fr (K.F.C.-J.); irenakrga@yahoo.com (I.K.); christine.morand@inra.fr (C.M.); 4 Institute for Biological Research “Siniša Stanković”—National Institute of Republic of Serbia, University of Belgrade, 11060 Belgrade, Serbia; avlada@ibiss.bg.ac.rs (V.A.); marko.miler@ibiss.bg.ac.rs (M.M.); verica.milosevic@gmail.com (V.M.); 5 Nutrigenomics Research Group, Departament de Bioquímica i Biotecnologia, Universitat Rovira i Virgili, 43007 Tarragona, Spain; anna.arola@urv.cat (A.A.-A.); franciscaisabel.bravo@urv.cat (F.I.B.); manuel.suarez@urv.cat (M.S.); 6 Department of Medicine, Democritus University of Thrace, Dragana, 68100 Alexandroupolis, Greece; edeligia@med.duth.gr (G.-E.D.); ckontogi@med.duth.gr (C.K.); 7 Department of Food Science, Czech University of Life Sciences, 16521 Prague, Czech Republic; jaroslav.havlik@gmail.com; 8 Institute of Biology and Immunology of Reproduction, Bulgarian Academy of Sciences, 1113 Sofia, Bulgaria; kistanova@gmail.com; 9 Centre of Research Excellence in Nutrition and Metabolism, Institute for Medical Research, National Institute of Republic of Serbia, University of Belgrade, 11060 Belgrade, Serbia; 10 National Research Council (CNR) Institute of Clinical Physiology (IFC), 73100 Lecce, Italy; marika@ifc.cnr.it (M.M.); egeria.scoditti@ifc.cnr.it (E.S.); 11 Norwich Medical School, University of East Anglia, Norwich NR4 7TJ, UK; d.vauzour@uea.ac.uk; 12 Department of Internal Medicine, Division of Cardiovascular Medicine, School of Medicine, University of California Davis, Davis, CA 95616, USA

**Keywords:** systematic literature search, nutrigenomics, integrative bioinformatics, polyphenols, cardiometabolic health, human

## Abstract

Cardiometabolic disorders are among the leading causes of mortality in the human population. Dietary polyphenols exert beneficial effects on cardiometabolic health in humans. Molecular mechanisms, however, are not completely understood. Aiming to conduct in-depth integrative bioinformatic analyses to elucidate molecular mechanisms underlying the protective effects of polyphenols on cardiometabolic health, we first conducted a systematic literature search to identify human intervention studies with polyphenols that demonstrate improvement of cardiometabolic risk factors in parallel with significant nutrigenomic effects. Applying the predefined inclusion criteria, we identified 58 differentially expressed genes at mRNA level and 5 miRNAs, analyzed in peripheral blood cells with RT-PCR methods. Subsequent integrative bioinformatic analyses demonstrated that polyphenols modulate genes that are mainly involved in the processes such as inflammation, lipid metabolism, and endothelial function. We also identified 37 transcription factors that are involved in the regulation of polyphenol modulated genes, including RELA/NFKB1, STAT1, JUN, or SIRT1. Integrative bioinformatic analysis of mRNA and miRNA-target pathways demonstrated several common enriched pathways that include MAPK signaling pathway, TNF signaling pathway, PI3K-Akt signaling pathway, focal adhesion, or PPAR signaling pathway. These bioinformatic analyses represent a valuable source of information for the identification of molecular mechanisms underlying the beneficial health effects of polyphenols and potential target genes for future nutrigenetic studies.

## 1. Introduction

Polyphenols are plant secondary metabolites with important physiological functions [1,2] and their production is increased as a response to external stressors, such as drought, cold, heat, UV irradiation, to cite only a few [3]. More than 8000 different polyphenols have been described in planta [4], but only about 500 are relevant for human nutrition [5]. Dietary polyphenols are divided into flavonoids and non-flavonoids. The subclass of flavonoids is further subdivided into flavanols, flavonols, flavones, isoflavones, flavanones, and anthocyanins. Nutritionally relevant non-flavonoids include phenolic acids, hydroxycinnamates, stilbenes, and lignans [1]. It is estimated that the usual human diet provides an average daily intake of approximately 1 g of total polyphenols [6]. However, there is a high variation in the daily intake of polyphenols, as it can range from less than 500 [7] to more than 1500 mg/day [8], which reflects the differences in nutritional habits between individuals, but also at the population level.

Epidemiological studies have reported positive effects of polyphenol intake on cardiometabolic health in humans [9,10]. These data initiated numerous human intervention trials to study the effects of various dietary polyphenols on cardiometabolic risk factors in subjects of different gender, age, or health status. Interventions included pure compounds and plant extracts along with various polyphenol-rich foods or beverages. Given the diversity in study populations, along with food bioactives and food matrices, the variability observed in the different outcomes is not surprising. The vast majority of studies report beneficial effects of polyphenols on cardiometabolic risk factors. For example, it has been demonstrated that cocoa polyphenols improve endothelial function in patients with non-alcoholic steatohepatitis [11]. In hypertensive patients with impaired glucose tolerance, cocoa polyphenols improved endothelial function and insulin sensitivity and decreased systolic and diastolic blood pressure [12]. A positive effect on blood pressure has also been reported with grape seed extract treatment in subjects with pre-hypertension [13]. In addition, grape polyphenols prevented fructose-induced oxidative stress and insulin resistance in overweight or obese first-degree relatives of patients with type 2 diabetes [14]. Extra virgin olive oil polyphenols also have been suggested to have a positive effect on cardiometabolic health in humans [15,16]. Furthermore, several studies have revealed within-group variability in response to the intake of these plant food bioactives [17,18]. Analyses of interindividual variability in response to dietary polyphenols identified potential main factors involved, such as gender, age, ethnicity, disease, and metabolic state, gut microbiota, and gene polymorphisms [19,20].

Although there are a lot of studies pointing to the direction of beneficial effects of polyphenols on cardiometabolic health in humans, as yet no clear molecular mechanisms have been clearly highlighted. Several studies have shown that diets, foods, and drinks (such as Mediterranean diet or low-dose alcohol consumption) can exert important nutrigenomic modifications which present important molecular mechanisms underlying their health properties [21,22]. Notwithstanding, experimental evidence indicates that many of the biological effects of polyphenols are mediated through nutri(epi)genomic mechanisms involving interactions with cell signaling proteins and transcription factors (TFs) [23]. For example, anthocyanins and their metabolites possess the capacity to bind with signaling proteins that are involved in monocyte adhesion and trans-endothelial migration, processes that are attenuated in vitro following pretreatment of endothelial cells with these bioactive compounds and their circulating metabolites [24]. Furthermore, integrative systematic bioinformatic analyses in cell models relevant for cardiometabolic disease, which include adipocytes, hepatocytes, endothelial, smooth muscle, and immune cells, demonstrated that flavanols and their metabolites modulate the expression of genes that are predominantly involved in inflammation, leukocyte adhesion and trans-endothelial migration, and lipid metabolism [25]. Polyphenols may modulate the expression/activity of the enzymes of arachidonic acid cascade and consequently the biosynthesis of eicosanoids, thereby controlling the inflammatory processes involved in cardiovascular diseases [26]. Polyphenols can also interact with specific receptors, such as type 2 taste receptors, which are responsible for the detection of bitter taste. These receptors have also been discovered in several extra-oral tissues, including human intestine where they have been associated with nutrient-gut interactions that influence gastrointestinal motility, appetite, and glycemia [27].

It is particularly difficult to investigate molecular mechanisms underlying positive cardiometabolic effects of polyphenols in humans, and therefore, the number of such studies is limited. Recruitment of participants with similar baseline characteristics, compliance with the intervention and participants’ follow-up in long-term studies, are all very challenging. Availability of samples for analyses is another limiting factor in human intervention studies, most of them being conducted using blood samples for analysis of gene expression, and only a small number of studies included skeletal muscle or adipose tissue [28,29]. Although numerous human intervention studies analyzed the effects of polyphenols on cardiometabolic risks, such as blood lipids, blood pressure, blood glucose, insulin resistance, endothelial function, oxidative stress, etc., many have not assessed the effects on gene expression. Another challenge in studying the molecular mechanisms of polyphenols’ health properties is the use of different methodological approaches in investigating the gene expression. For example, apart from the classical methodology of analyzing gene expression of several target RNAs with RT-PCR methods, innovative -omics technologies allow analysis of gene expression of hundreds or thousands of genes at the same time. In terms of the quantity of obtained data, the advantage of the -omics technologies is undoubtable. However, the fact that verification with classical methods does not always corroborate with the obtained results [30,31], imposes the need for separate analyses and interpretation of data from studies that had adopted different methodological approaches.

Therefore, we conducted a systematic literature search aiming to identify human intervention studies with polyphenols that demonstrated significant modulation in gene expression in parallel with positive effect/s on cardiometabolic risk factors. In our study, we focused only on studies that adopted a targeted approach, i.e., analyzed changes in the expression of several target mRNAs or miRNAs with RT-PCR methods. We then applied integrative bioinformatic analyses of identified nutrigenomic effects, to gain a better understanding of the overall molecular mechanisms of polyphenols in humans underlying their health properties.

## 2. Materials and Methods

### 2.1. Strategy for Literature search and Data Extraction

All literature relevant to the effect of polyphenols on gene expression and cardiometabolic endpoints in human trials was searched and obtained using the Preferred Reporting Items for Systematic Reviews (PRISMA) statement guidelines with a predetermined search strategy [32]. A comprehensive search on PubMed and Web of Science, using Medical Subject Headings (MeSH) and Boolean operators where appropriate, was conducted in July 2018, with an update in November 2019. The search included keywords referring to bioactives (polyphenols, flavonoids, flavanols, flavanones, epicatechin, catechin, procyanidin, anthocyanins, resveratrol, hydroxytyrosol, extracts, fruits, juice, grapes, citrus, pomegranate, apple, tea, coffee, cocoa, olive oil, chocolate, berries, isoflavones, daidzein, equol, hesperetin), type of studies (human, clinical trials, randomized, patients, volunteers, males, females), nutrigenomic analysis (nutrigenomic, genomic, genome, gene, gene expression, transcription, mRNA, messenger RNA, RT-PCR, PCR-arrays, microarrays, macroarrays, epigenome, miRNA, ncRNA), and target tissues (peripheral blood mononuclear cells, T-cells, B-cells, lymphocytes, monocytes, blood, blood cells, platelets, adipose tissue, adipocytes, liver, plasma, serum). Following the identification of the publications using the aforementioned terms, the search results were narrowed down by only selecting studies with a targeted approach published in English language.

Inclusion criteria for data extraction were as follows: human intervention studies of cardiometabolic disease or cardiometabolic risk factors where polyphenols were used for intervention, and modulation of gene expression was studied at mRNA and/or miRNA level, which was analyzed with RT-PCR methods. Furthermore, in order to be included in our analysis, the publications should have reported at least one beneficial cardiometabolic outcome, such as: improved plasma lipid status (total cholesterol, triglycerides, HDL-cholesterol, LDL-cholesterol, apoA1, apoB, or oxLDL, etc.), improved oral glucose tolerance test, decreased fasting glucose, glycated hemoglobin, insulin resistance, blood pressure, body weight, waist circumference, systemic inflammation (circulating CRP, TNFα, interleukins, etc.), or decreased oxidative stress. Studies reporting an unfavorable cardiometabolic outcome, for example, increased LDL-cholesterol, were excluded from our analysis. In addition, studies that did not include a control of polyphenol intervention, or studies of co-interventions (for example, co-interventions of polyphenols with vitamins or exercise), were also excluded.

A template for data extraction was designed specifically for this study. The template was pilot tested to verify the accuracy of the data extraction. The final version of the template was distributed among the participants. Extracted data include: details about the paper (PMID, authors, year of publication, title), information about the study population (gender, age, number of participants, health status), information about the intervention (bioactive/s, dose and duration of intervention), study design, information about the cardiometabolic outcomes (including both studied and significantly modulated), and information about gene expression (mRNA and/or miRNA, analytical method, tissue/cells analyzed for gene expression, gene symbol as reported in the paper, official gene symbol, and official gene name). Only genes that were significantly modulated by the intervention (*p* < 0.05) were extracted from the eligible papers, and subsequently subjected to bioinformatic analyses. Extracted data were cross-checked by two participants. In case of doubts or disagreement, a third participant was consulted.

### 2.2. Bioinformatic Analyses

Pathways enrichment analyses were conducted using GeneTrail2 (https://genetrail2.bioinf.uni-sb.de/, accessed on 24 January 2020) [33], version 1.6, as a platform to access KEGG and BioCarta databases, using the following settings: over-representation analysis; null hypothesis (for *p*-value computation)—two-sided; method to adjust *p*-values—Benjamini-Yekutieli; significance level—0.05. All supported genes in the NCBI database were taken as a reference.

Interactions between functional groups of genes were analyzed with Cytoscape software (version 3.7.2; https://cytoscape.org/, accessed on 1 June 2020) [34], using the application ClueGO [35], connected to CluePedia [36].

Protein-protein interactions (PPIs) were analyzed using the database STRING (https://string-db.org/, accessed on 25 January 2020) [37], version 11.0, applying the following settings: confidence; text-mining, experiments, databases, co-expression; high confidence—0.700; no more than 10 interactions in the first shell and no more than 5 interactions in the second shell. The obtained protein network was organized in three clusters of functionally interconnected proteins.

Transcription factors that regulate the expression of polyphenol modulated genes were identified using Enrichr (https://amp.pharm.mssm.edu/Enrichr/, accessed on 25 January 2020) [38,39], as a platform to access the database TRRUST Transcription Factors 2019 [40]. Interactions between the transcription factors and the genes they regulate were visualized with Cytoscape software (version 3.7.1; https://cytoscape.org/, accessed on 1 June 2020) [41].

To identify mRNAs regulated by polyphenol modulated miRNAs, i.e., predicted miRNA targets, we used miRBase (http://www.mirbase.org/, accessed on 8 February 2020) [42], as a platform to access the following databases: TargetMiner, TargetScanVert and miRDB. For ID conversion of genes that were retrieved with the database TargetMiner, we used the ID convert tool of the database SYNGO (https://syngoportal.org/, accessed on 8 February 2020) [43]. InteractiVenn (http://www.interactivenn.net/, accessed on 8 February 2020) [44] was used as a tool to retrieve the predicted miRNA targets that are in common for the selected databases, which were subsequently used for pathways enrichment and integration analyses. Interactions between miRNA targets were visualized with Cytoscape software (version 3.7.1; https://cytoscape.org/, accessed on 1 June 2020) [41]. To compare the mRNAs extracted from the literature to the miRNA targets, and to analyze (a) the genes in common and (b) the genes that belong to the same enriched (ontology) term, we used the bioinformatic tool Metascape (http://metascape.org, accessed on 3 April 2020; the option “Multiple Gene List”) [45], where the results are visualized with Circos plot (http://circos.ca/, accessed on 3 April 2020) [46]. Enriched terms for both gene sets are visualized using a heatmap plot.

To compare KEGG pathways associated with mRNAs extracted from the literature and predicted miRNA targets, the enriched pathways obtained in our previous steps were used to build a network of pathways; two pathways were considered interconnected where at least one of the mRNAs or miRNA targets involved in them are common to both. Networks were constructed and visualized using Cytoscape software (version 3.7.1; https://cytoscape.org/, accessed on 1 June 2020) [41]. Data preparation was performed with the use of several R packages, including splitstackshape (https://github.com/mrdwab/splitstackshape, accessed on 16 June 2020), data.table (https://github.com/Rdatatable/data.table, accessed on 16 June 2020), dplyr (http://dplyr.tidyverse.org, accessed on 16 June 2020; https://github.com/tidyverse/dplyr, accessed on 16 June 2020), and string (http://stringr.tidyverse.org, accessed on 16 June 2020; https://github.com/tidyverse/stringr, accessed on 16 June 2020). Pathway networks were built separately for pathways enriched in each category and pathways considering all categories’ components together in a global pathway enrichment analysis. To obtain the pathways with the highest degree (number of connections of one node to other nodes), the Cytoscape Network Analyzer application was used (http://apps.cytoscape.org/apps/networkanalyzer, accessed on 16 June 2020).

For integrative analysis of extracted mRNAs, their associated TFs, and extracted miRNAs, we used the 3D-layer option in the bioinformatic tool OmicsNet (https://www.omicsnet.ca/, accessed on 10 February 2020) [47,48]. Official gene names and symbols were identified using GeneCards (https://www.genecards.org/, accessed on 22 May 2020) [49]. Where applicable, protein names were searched in the UniProt database (https://www.uniprot.org/, accessed on 22 May 2020) [50].

## 3. Results

### 3.1. Studies, Bioactives and Differentially Expressed Genes

**Figure 1:**
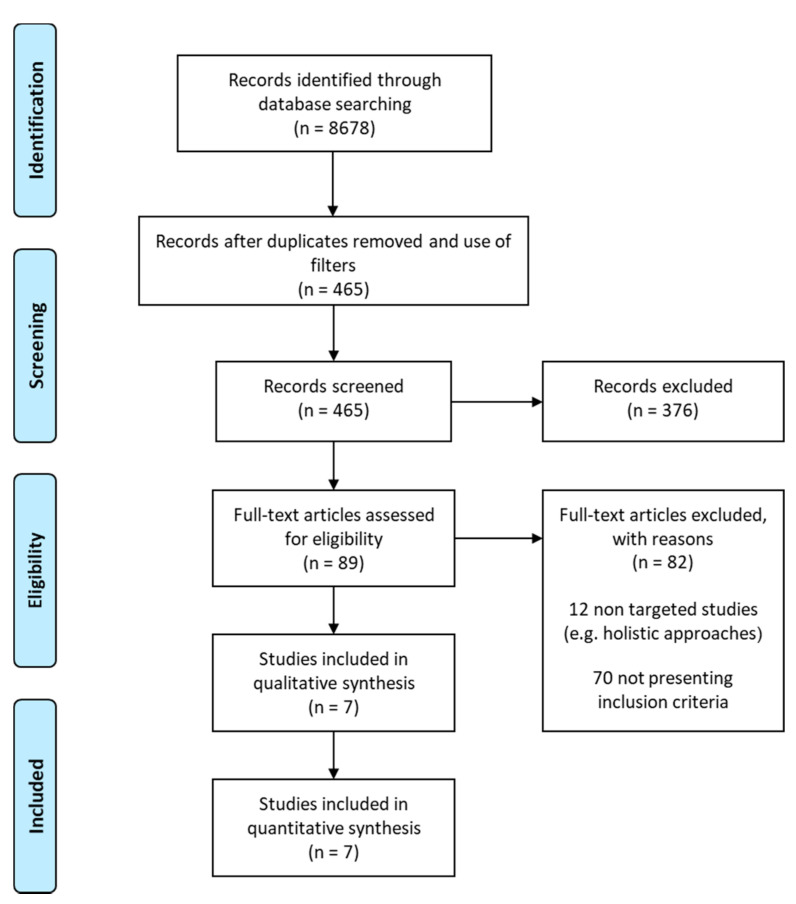
Flowchart of the literature search and data extraction.

A comprehensive search on PubMed and Web of Science, conducted in July 2018, with an update in November 2019, resulted in 8678 documents. After removal of duplicates, and using “Human” and “RCT” and “Clinical study” and “Clinical trial” and “Controlled Clinical Trial” and “Multicenter Study” filters, this number dropped to 465 manuscripts, which were further screened for eligibility. Based on titles and abstracts, 89 papers were selected for data extraction and distributed among the participants (Figure 1). After detailed analysis of the full text, and applying the predefined inclusion/exclusion criteria, we identified seven papers that report significant changes in gene expression accompanied with at least one favorable cardiometabolic outcome in controlled human intervention studies with polyphenols [51,52,53,54,55,56,57]. For one of the studies [57], information about the study design and cardiometabolic outcomes was extracted from a previously published paper [58].

All studies used peripheral blood mononuclear cells (PBMCs), except one in which white blood cells were used, as biological material for analysis of modulations in gene expression (Table 1). Polyphenols from different origins, including olive oil and grape extracts, as well as pure compounds such as resveratrol, quercetin, or curcumin were used in the nutrigenomic studies. Doses varied in a wide range, as did the duration of interventions, which ranged from 2 or 5 h (acute studies) up to one year (chronic studies). Study populations included adults (men, women or both) of various age groups, up to 80 years old. There was also a large variability in the health status of study populations, ranging from healthy subjects with cardiometabolic risk factors, to patients with a diagnosed cardiometabolic disease (Table 1). As for the modulation of gene expression, we identified 58 differentially expressed genes at mRNA level (one duplicate was removed), and 5 differentially expressed miRNAs (Table 1), which were subjected to bioinformatic analyses.

### 3.2. mRNAs—Bioinformatic Analyses

#### 3.2.1. Pathways Analyses

**Figure 2:**
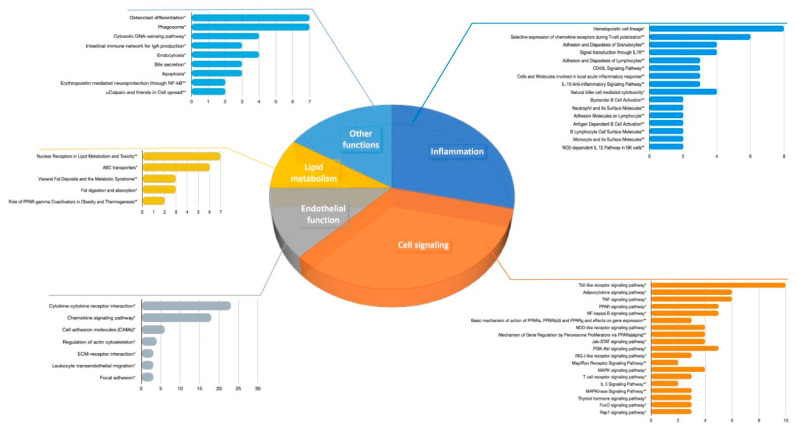
KEGG and top BioCarta pathways related to cellular processes, significantly associated with polyphenol modulated mRNA transcripts (*x*-axis represents the number of hits, i.e., number of genes in the pathway that are extracted from the eligible papers; within each group, pathways are arranged according to their *p*-values, in ascending order). KEGG pathways are marked with *; BioCarta pathways are marked with **.

To better understand the biological meaning of genes extracted from the literature, we first conducted bioinformatic analyses to identify pathways significantly associated with polyphenol modulated genes. Using GeneTrail2 as a platform to access KEGG and BioCarta databases, we identified N = 71 KEGG and N = 42 BioCarta pathways that are significantly associated with extracted genes, modulated at the mRNA level. Among KEGG pathways retrieved with this analysis, N = 32 pathways are related to cellular processes, whereas the others are related to various diseases. Among the top 25 BioCarta pathways, N = 24 are related to cellular processes. A deeper insight into KEGG and BioCarta pathways related to cellular processes demonstrated that these pathways are mainly involved in endothelial function, cell signaling, inflammation, and lipid metabolism, as depicted in Figure 2. The most enriched pathways include: cytokine-cytokine receptor interaction, encompassing N = 23 differentially expressed genes at mRNA level, i.e., hits, (*CCL20*, *CCL22*, *CCL3*, *CCR1*, *CCR5*, *CCR7*, *CD40*, *CD40LG*, *CX3CR1*, *CXCL10*, *CXCL16*, *CXCL6*, *CXCL9*, *CXCR1*, *CXCR2*, *CXCR3*, *CXCR6*, *IL1B*, *IL1R2*, *IL23A*, *IL6*, *IL7R*, *TNF*); chemokine signaling pathway (with N = 18 hits: *CCL20*, *CCL22*, *CCL3*, *CCR1*, *CCR5*, *CCR7*, *CX3CR1*, *CXCL10*, *CXCL16*, *CXCL6*, *CXCL9*, *CXCR1*, *CXCR2*, *CXCR3*, *CXCR6*, *NFKBIA*, *RAC1*, *STAT1*); cell adhesion molecules (N = 6 hits: *CD40*, *CD40LG*, *ICAM2*, *ICAM3*, *ITGB2*, *PECAM1*); toll-like receptor signaling pathway (N = 10 hits: *CCL3*, *CD40*, *CXCL10*, *CXCL9*, *IL1B*, *IL6*, *NFKBIA*, *RAC1*, *STAT1*, *TNF*); adipocytokine signaling pathway (N = 6 hits: *ADIPOR1*, *ADIPOR2*, *CD36*, *NFKBIA*, *PPARA*, *TNF*); TNF signaling pathway (N = 6 hits: *CCL20*, *CXCL10*, *IL1B*, *IL6*, *NFKBIA*, *TNF*); PPAR signaling pathway (N = 5 hits: *CD36*, *OLR1*, *PPARA*, *PPARD*, *PPARG*); NF-kappa B signaling pathway (N = 5 hits: *CD40*, *CD40LG*, *IL1B*, *NFKBIA*, *TNF*); PI3K-Akt signaling pathway (N = 5 hits: *IL6*, *IL7R*, *ITGA5*, *ITGB3*, *RAC1*); nuclear receptors in lipid metabolism and toxicity (N = 7 hits: *ABCA1*, *ABCB4*, *ABCC2*, *ABCG1*, *PPARA*, *PPARD*, *PPARG*); ABC transporters (N = 6 hits: *ABCA1*, *ABCA2*, *ABCA4*, *ABCB4*, *ABCC2*, *ABCG1*); hematopoietic cell lineage (N = 8 hits: *CD36*, *IL1B*, *IL1R2*, *IL6*, *IL7R*, *ITGA5*, *ITGB3*, *TNF*); selective expression of chemokine receptors during T-cell polarization (N = 6 hits: *CCL3*, *CCR1*, *CCR5*, *CCR7*, *CD40LG*, *CXCR3*).

#### 3.2.2. Interactions between Functional Groups of Genes

**Figure 3:**
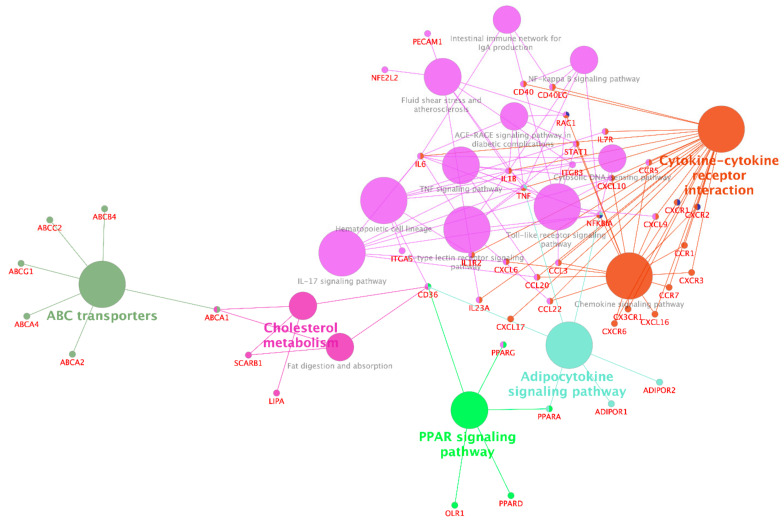
Interactions between functional groups of genes modulated by polyphenols.

Analysis of interactions between functional groups of differentially expressed genes was conducted using the applications ClueGO and CluePedia in Cytoscape software, calculating not only the interactions between polyphenol-affected pathways, but also the role and involvement of modulated genes within the network. Similar to pathways enrichment analyses, disease-related pathways were excluded from the graphical presentation (Figure 3). Analysis of interactions between functional groups of genes confirms the predominant effects of polyphenols on pathways involved in cell signaling related to inflammation, endothelial dysfunction, and lipid metabolism. In addition, this analysis demonstrates that *TNF* and *NFKBIA* are shared among at least three of the identified clusters of pathways, depicted in different colors in Figure 3. As such, these genes are likely to play a central role in cardiometabolic health-promoting effects of polyphenols.

#### 3.2.3. Protein-Protein Interactions

**Figure 4:**
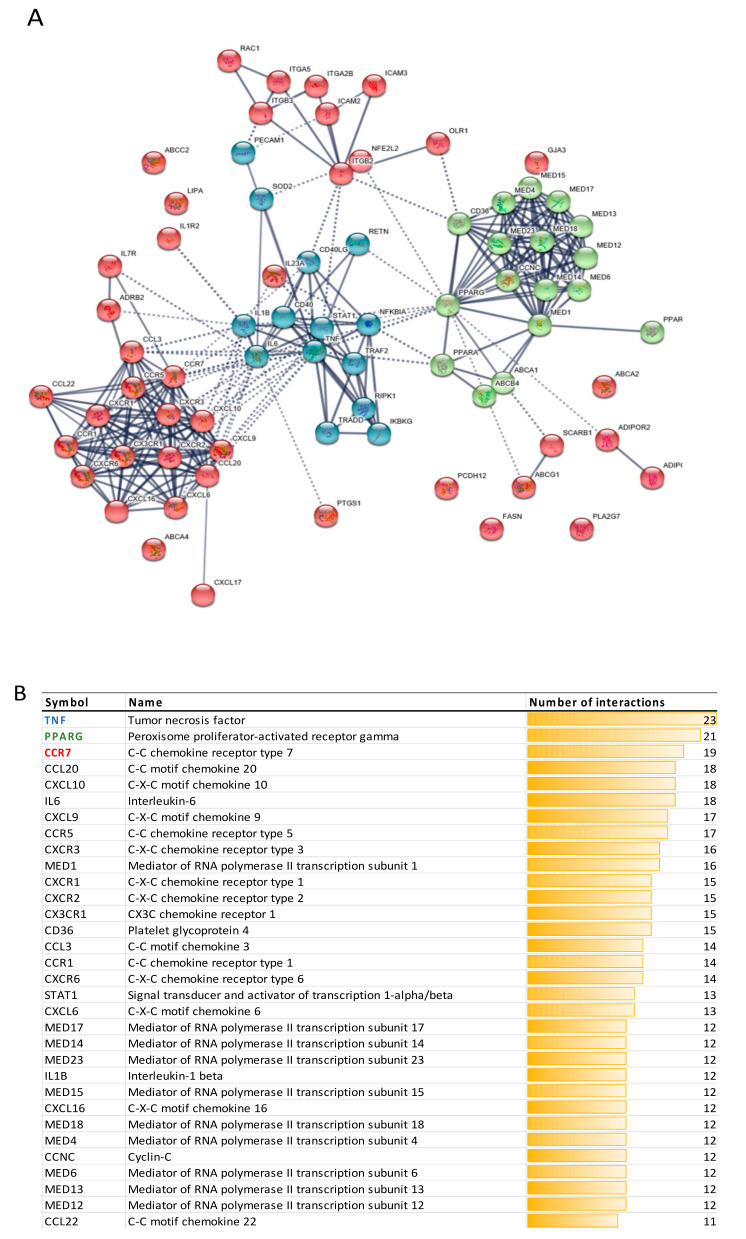
(**A**) Protein-protein interactions for proteins encoded by the extracted genes, as well as several neighboring proteins (up to 10 interactions within the first shell; up to 5 interactions within the second shell), organized in 3 clusters. (**B**) Proteins that have more than 10 interactions within the protein-protein interactions network. Top 3 proteins belong to a different cluster each.

Furthermore, we performed protein-protein interactions analysis for proteins encoded by polyphenol modulated mRNA transcripts, using the bioinformatic tool STRING. This analysis also revealed N = 15 neighboring proteins, ten of which were within the first shell of interactions, and five within the second shell. We also used the functionality of STRING for the organization of proteins in functionally differentiated clusters, which allows better interpretation of extracted data. For our set of differentially expressed genes, three well-defined protein clusters are clearly distinctive (Figure 4A), mainly involved in inflammation (blue color), PPAR signaling (green color), and chemokine signaling (most of the proteins in red color). Figure 4A also clearly demonstrates that some of the proteins have more interactions within the network than the others. Proteins that have more than 10 interactions are presented in Figure 4B. On top of the list are the hub proteins for each cluster: TNF with 23 interactions (blue), PPARG with 21 interactions (green), and CCR7 with 19 interactions (red).

#### 3.2.4. Transcription Factors

**Figure 5:**
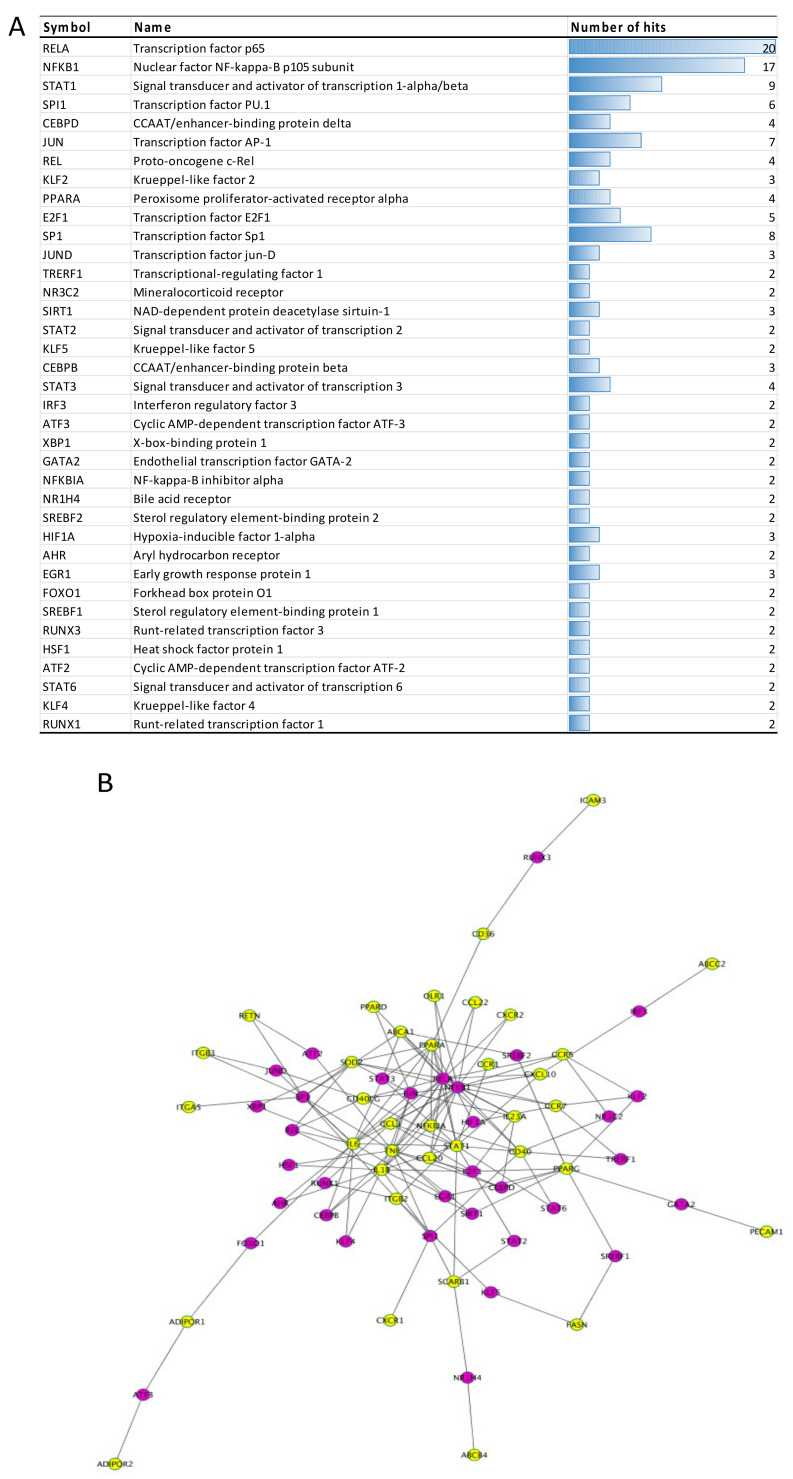
Regulation of differentially expressed mRNAs at the transcriptional level. (**A**) Predicted transcription factors that regulate polyphenol modulated genes, arranged according to their *p*-values, in ascending order. (**B**) Interconnections between predicted transcription factors (in purple color) and polyphenol modulated genes (in yellow color).

We also aimed to elucidate which transcription factors could have their activity modulated by polyphenols and be involved in the regulation of the expression of genes extracted from the literature. Using the bioinformatic tool Enrichr to access the database TRRUST Transcription Factors 2019, we identified N = 37 transcription factors that are significantly associated with our set of mRNAs (adjusted *p*-value < 0.05). The top 5 transcription factors retrieved with this analysis include: RELA and NFKB1 with more than 15 hits each, STAT1 with 9 hits, SPI1 and CEBPD (Figure 5A). Interconnections between predicted transcription factors and polyphenol modulated genes extracted from the literature are depicted in Figure 5B.

### 3.3. miRNAs—Bioinformatic Analyses

#### 3.3.1. miRNA Targets

**Figure 6:**
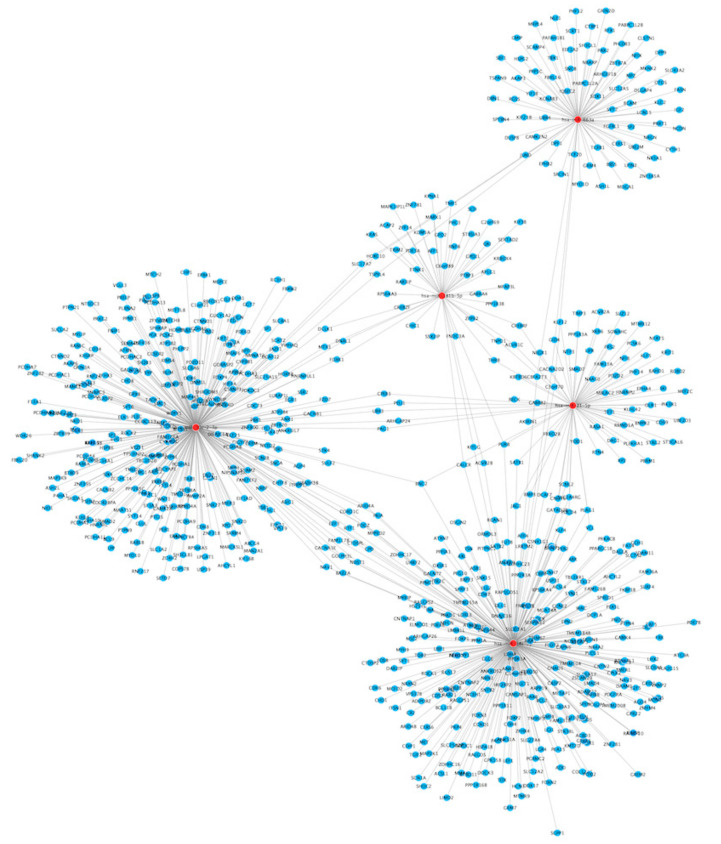
miRNAs modulated by polyphenols (red circles), their target mRNAs (blue circles), and interactions.

Whilst analyzing the eligible papers for data extraction, we identified N = 5 miRNAs which expression was modulated by polyphenols (Table 1). To elucidate their biological functions, we first aimed to retrieve their target mRNAs. To this aim, for each miRNA, their targets were mapped in 3 databases (TargetMiner, TargetScanVert, and miRDB), which were accessed through miRBase. For each miRNA, the targets that were in common for all 3 databases were considered for further analyses, such as pathways enrichment analyses and integration analyses. Using this approach, we obtained: hsa-miR-21-5p:74 targets; hsa-miR-181b-5p:49 targets; hsa-miR-663a:81 targets; hsa-miR-30c-2-3p:290 targets; hsa-miR-34a-5p:278 targets. Interactions between miRNAs and their predicted target mRNAs are presented in Figure 6.

#### 3.3.2. Pathways Analyses of miRNA Targets

**Figure 7:**
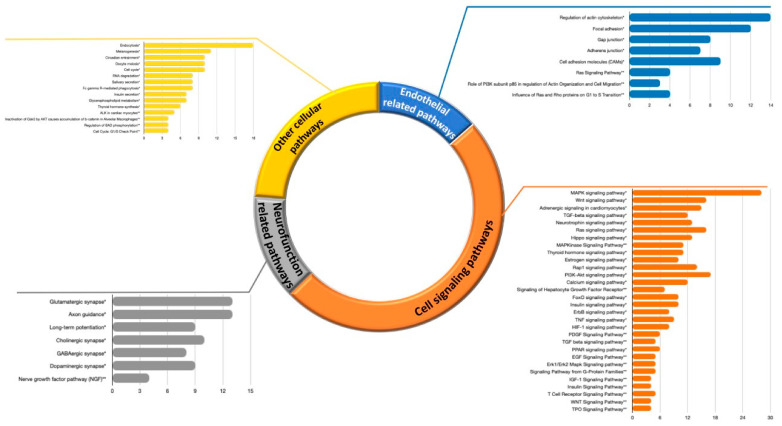
Top 60 KEGG and BioCarta pathways related to cellular processes, significantly associated with predicted miRNA targets (*x*-axis represents the number of hits; within each group, pathways are arranged according to their *p*-values, in ascending order). KEGG pathways are marked with *; BioCarta pathways are marked with **.

We also conducted bioinformatic analysis of pathways that are significantly associated with the predicted miRNA targets. To this aim, targets of all 5 miRNAs were taken together, and duplicates were removed. In that way, 720 targets were identified and analyzed with GeneTrail2 for KEGG and BioCarta pathways. We identified in total N = 86 KEGG and N = 21 BioCarta pathways that are significantly associated with miRNA targets (adjusted *p*-value < 0.05). Some of these pathways are associated with cellular processes, whereas others are related to various diseases. A deeper insight into the top 60 pathways associated with cellular processes (Figure 7) demonstrated that the vast majority is involved in cell signaling, such as: MAPK signaling pathway (N = 28 hits: *CACNA1E*, *CACNA2D2*, *CACNB1*, *CACNB2*, *DUSP8*, *ELK1*, *HSPA1B*, *JUND*, *KRAS*, *MAP2K1*, *MAP4K1*, *MEF2C*, *MKNK2*, *NTF3*, *PAK1*, *PDGFRA*, *PPM1A*, *PPP3R1*, *PPP5C*, *PRKACB*, *RASA1*, *RPS6KA3*, *RPS6KA4*, *RPS6KA5*, *RRAS*, *STK4*, *TAOK1*, *TGFB1*), Wnt signaling pathway (N = 16 hits: *CAMK2A*, *CCND1*, *CHD8*, *CTBP1*, *DAAM1*, *FZD4*, *FZD7*, *LEF1*, *NKD1*, *PPP3R1*, *PRKACB*, *PSEN1*, *ROCK2*, *SMAD4*, *TBL1XR1*, *WNT1*), Ras signaling pathway (N = 16 hits: *EFNA1*, *ELK1*, *KITLG*, *KRAS*, *MAP2K1*, *MET*, *PAK1*, *PDGFRA*, *PIK3R1*, *PLCG1*, *PRKACB*, *RAB5B*, *RALGDS*, *RASA1*, *RRAS*, *STK4*), or PI3K-Akt signaling pathway (N = 17 hits: *BCL2*, *CCND1*, *CCNE2*, *CDK6*, *CREB3L1*, *EFNA1*, *KITLG*, *KRAS*, *MAP2K1*, *MET*, *PDGFRA*, *PHLPP2*, *PIK3R1*, *PPP2R3A*, *PPP2R5C*, *RELN*, *YWHAQ*).

Some of the pathways are involved in the regulation of endothelial function, such as: regulation of actin cytoskeleton (N = 14 hits: *ARPC2*, *CYFIP2*, *IQGAP3*, *KRAS*, *MAP2K1*, *MYH9*, *PAK1*, *PDGFRA*, *PIK3R1*, *PIP5K1A*, *ROCK1*, *ROCK2*, *RRAS*, *VCL*), focal adhesion (N = 12 hits: *BCL2*, *CCND1*, *ELK1*, *MAP2K1*, *MET*, *PAK1*, *PDGFRA*, *PIK3R1*, *RELN*, *ROCK1*, *ROCK2*, *VCL*), cell adhesion molecules (N = 9 hits: *CD34*, *CDH4*, *CNTN2*, *CNTNAP1*, *CNTNAP2*, *MPZ*, *NEGR1*, *NFASC*, *NRXN2*), gap junction (N = 8 hits: *GUCY1A2*, *HTR2C*, *ITPR2*, *KRAS*, *MAP2K1*, *PDGFRA*, *PRKACB*, *PRKG2*), or adherens junction (N = 7 hits: *CTNND1*, *LEF1*, *MET*, *SMAD2*, *SMAD4*, *SSX2IP*, *VCL*). Interestingly, some of the top pathways are also related to the function of the nervous system such as: axon guidance (N = 13 hits: *EFNA1*, *EPHA4*, *EPHB2*, *KRAS*, *MET*, *PAK1*, *PLXNA2*, *PPP3R1*, *RASA1*, *ROBO2*, *ROCK1*, *ROCK2*, *SEMA4G*), or glutamatergic synapse (N = 13 hits: *GLUL*, *GNAO1*, *GRIN2D*, *GRIN3A*, *GRM4*, *GRM7*, *HOMER1*, *ITPR2*, *PPP3R1*, *PRKACB*, *SHANK2*, *SLC17A7*, *SLC1A2*).

### 3.4. Integration Analyses

#### 3.4.1. Integration of mRNAs and miRNA Targets

**Figure 8:**
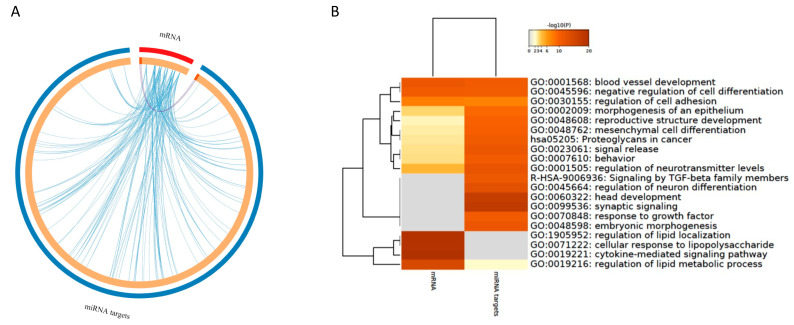
Comparative analysis of mRNAs extracted from the literature and the predicted miRNA targets. (**A**) Overlapping genes (purple curves) and genes that share the same enriched term (blue curves). (**B**) Heatmap of enriched terms, colored by *p*-values.

Comparative analysis of mRNAs extracted from the literature and the predicted miRNA targets was conducted using the bioinformatic tool Metascape. This analysis demonstrated that within these two gene sets, there are only three genes that overlap, namely *FASN*, *ADIPOR2*, and *OLR1* (presented with purple curves in Figure 8A). Despite the small number of overlapping genes, however, this analysis retrieved a remarkable number of functional interactions between these two gene sets, demonstrated with a number of genes that share the same enriched term (blue curves in Figure 8A). Detailed information about the genes from both gene sets that belong to the top 20 enriched terms is presented in Table S1 in the Supplementary Materials. Although there are terms that are enriched exclusively for one of the analyzed gene sets, such as regulation of lipid localization, cellular response to lipopolysaccharide or cytokine-mediated signaling pathway for mRNAs, or signaling by TGF-beta family members for miRNA targets, the heatmap of enriched terms demonstrates that these two gene sets share several important gene ontologies, including regulation of cell adhesion or signal release (Figure 8B).

#### 3.4.2. Integration of mRNA and miRNA-Target Pathways

**Figure 9:**
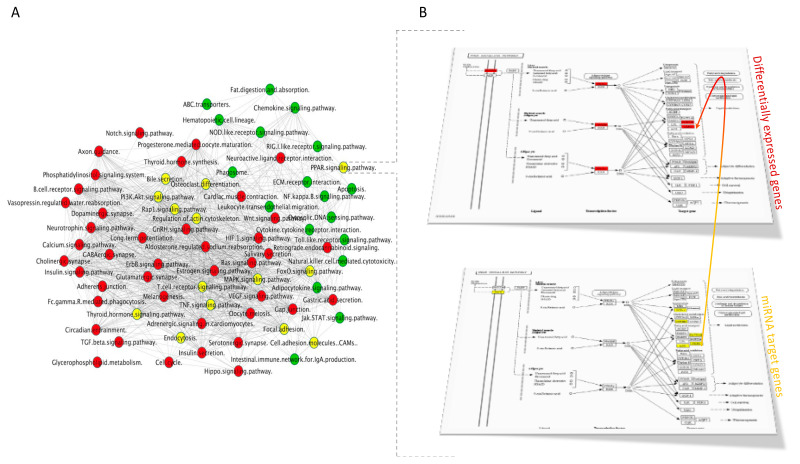
(**A**) mRNAs and miRNA-targets network of pathways. List of differentially expressed genes and miRNA targets were used to perform a global GeneTrail2 enrichment analysis and identify enriched pathways. A pathway-connections network was built in Cytoscape. Node colored labels represent the categories (mRNA, miRNA targets or combination) from which the pathways were enriched. Red circles are miRNA pathways, yellow are miRNA and mRNA pathways, green are mRNA pathways. (**B**) PPAR signaling pathway in 2 levels, one for mRNA and one for miRNA targets.

The global network of enriched pathway interactions of our study categories (mRNAs and miRNA targets) was grouped and shown in Figure 9A, where individual clusters were not differentiated, but rather a centralized network. The pathways with the highest degree of connections with other pathways into the network include MAPK signaling pathway, thyroid hormone signaling pathway, TNF signaling pathway, estrogen signaling pathway, Rap1 signaling pathway, and gap junction. From this integration analysis, we identified common enriched pathways to mRNAs and miRNA targets that include MAPK signaling pathway, thyroid hormone signaling pathway, TNF signaling pathway, PI3K-Akt signaling pathway, T cell receptor signaling pathway, focal adhesion, regulation of actin cytoskeleton, cell adhesion molecules (CAMs), PPAR signaling pathway. Regarding PPAR signaling pathway, it has been identified as significantly over-represented with 5 differentially expressed genes (*CD36*, *OLR1*, *PPARA*, *PPARD*, *PPARG*) but also with 6 genes (*ACSL1*, *ACSL4*, *CYP8B1*, *OLR1*, *SCD*, *SLC27A4*) that are targets of miRNAs. Among these genes, *OLR1* was identified both as differentially expressed and target of hsa-miR-21-5p, suggesting the polyphenols by modulating expression of this miRNA can affect level of mRNA of *OLR1* (Figure 9B). This integrated analysis of differentially expressed genes identified using targeted approach across different studies shows complex and multi-gene mode of action of polyphenols, including both protein coding and non-coding genes involved in pathways that form a complex network that allow regulation of cellular functions.

#### 3.4.3. mRNAs, miRNAs and Transcription Factors Integration Analysis

**Figure 10:**
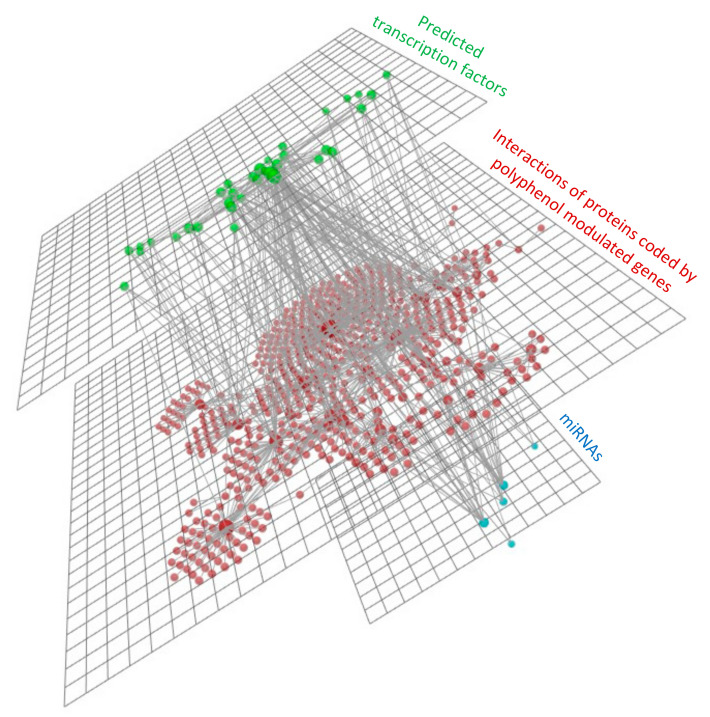
3D-layer presentation of interactions of proteins that are coded by polyphenol modulated genes (in red color), polyphenol modulated miRNAs (in blue color), and the predicted transcription factors (in green color).

To visualize interactions between polyphenol modulated genes (N = 58) and miRNAs (N = 5), as well as the predicted transcription factors (N = 37) that regulate the expression of polyphenol modulated genes, we used the bioinformatic tool OmicsNet. PPIs of proteins that are coded by polyphenol modulated genes are retrieved using the STRING database, and are presented in the middle of the 3D-layer presentation (Figure 10).

Transcription factors, retrieved using the TRRUST database, are presented above the PPIs, in green color. Modulated miRNAs by polyphenols are presented in blue color. Figure 10 clearly depicts the presence of numerous but specific interactions between these molecules, as determinants of the positive effects of polyphenols on cardiometabolic health in humans.

This study presents several limitations. We only used genomic data available from studies that used targeted gene expression analysis. There are several studies that have used untargeted genomic analysis, and provide significant information on genomic modifications by plant food bioactives. However, because of the large difference in number of differentially expressed genes identified due to approaches used, we did not include studies which used untargeted approaches. Moreover, this systematic analysis only included studies with positive effects on cardiometabolic endpoint analysis, however there are also studies that reported changes in the expression of specific genes related to other health effects.

## 4. Discussion

Cardiometabolic disorders are among the leading causes of mortality in human population and therefore attract much interest for finding effective solutions for prevention and treatment. Since diet and lifestyle are important factors influencing the onset and progression of cardiometabolic disorders, preventive measures are largely directed towards their modification. In terms of diet, polyphenols have an important role as plant food bioactives with protective effects. Namely, numerous epidemiological and human intervention studies indicate that polyphenols generally have positive effects on human cardiometabolic health [9,12]. Moreover, recent studies highlight the importance of interindividual variability in response to polyphenols intake, which is determined by many factors, including genetic variability. However, molecular mechanisms underlying health-promoting effects of polyphenols are not entirely clear, which is even more apparent for the potential influence of specific gene variants in humans. Aiming to clarify at least some of the molecular mechanisms that underlie the beneficial effects of polyphenols on cardiometabolic health, we conducted a systematic literature search followed by comprehensive bioinformatic analyses. The availability of data for significant modulation of both mRNAs and miRNAs allowed us to make an mRNA-miRNA integration, as well as integration at an additional, third level, analyzing the predicted transcription factors that regulate polyphenol modulated mRNAs.

Bioinformatic analyses of polyphenol modulated mRNAs demonstrated that these genes are mainly involved in processes such as inflammation, lipid metabolism and endothelial function. Network analyses pinpointed several genes with central positions within the functional clusters and the highest number of interactions, such as *TNF*, *NFKBIA*, *PPARG*, and *CCR7*. Among transcription factors, the most prominent role is demonstrated for RELA/NFKB1, STAT1, and JUN, but SIRT1, or KLF4 are also significantly associated. Most of these genes and molecules are important mediators of inflammatory response, which is the major underlying mechanism of cardiometabolic disorders [59]. Indeed, low-grade chronic inflammation, also referred to as metaflammation, has been identified as causative for obesity-induced insulin resistance [60], further progressing to atherogenic dyslipidemia, metabolic syndrome, type 2 diabetes and/or metabolic associated fatty liver disease (MAFLD) [61,62]. The process is initiated when obese, hypertrophied and dysfunctional adipocytes increase the secretion of free fatty acids and pro-inflammatory cytokines and activate adipose tissue-resident macrophages into pro-inflammatory M1 phenotype. M1 macrophages additionally release a variety of pro-inflammatory cytokines, including TNFα, IL1β and IL6, which act on adipocytes, liver and skeletal muscles, causing local and systemic insulin resistance, and oxidative stress [63,64]. The effects of these pro-inflammatory stimuli on insulin signaling are mediated through several inflammation-related kinases, including IKK and JNK. In addition to activation of NF-kappa B and AP1 respectively, leading to transcriptional activation of pro-inflammatory cytokines, these kinases are also involved in inhibitory serine phosphorylation of IRS, which results in inactivation of the PI3K-Akt pathway, impaired insulin signaling and insulin resistance [65]. This observation can be corroborated with studies that revealed the capacity of polyphenols to negatively regulate the NF-kappa B signaling pathway, depress the phosphorylation of kinases, inhibit NF-kappa B translocation into the nucleus but also interfere interactions between NF-kappa B and DNA [66]. It has also been shown, using NF-kappa B reporter gene assays, that monomeric and oligomeric flavanols from grape seeds can decrease the activity of this transcription factor [67]. The modulation of activity of transcription factors by polyphenols is probably mediated through interaction of these bioactives with cell signaling proteins. For example, we have previously shown that anthocyanins present high potential binding with cell signaling proteins like mTOR, FAK1, Smad2/3, MAPKs as JNK1/2/3 or MAP2K1 as well as IκB proteins [24]. These cell signaling proteins regulate the activity of transcription factors such as STAT1, JUN, SIRT1, NF-kappa B, or SPI1. Similar observation has been reported for epicatechin metabolites [68]. These results corroborate with other studies which showed that polyphenols can modify phosphorylation and activity of these cell signaling proteins and transcription factors [69,70]. Therefore, by interacting with cell signaling proteins, polyphenols will modulate their kinase activity, which in turn will affect activation of transcription factors and consequently expression of genes. Importantly, our bioinformatic analyses identified the NF-kappa B signaling pathway, along with adipocytokine signaling pathway, TNF signaling pathway, Toll-like receptor signaling pathway, and PI3K-Akt signaling pathway among the most significantly modulated by dietary polyphenols, demonstrating their anti-inflammatory and insulin-sensitizing mode of action.

The activity of NF-kappa B is also modulated by SIRT1, an NAD^+^ dependent deacetylase, which was identified in our bioinformatic analysis among the TFs significantly involved in the regulation of polyphenol modulated genes. In particular, SIRT1 has the ability to deacetylate the p65 subunit of NF-kappa B, thus inhibiting its transcriptional activity [71], which results in reduced inflammation in adipocytes and macrophages [72,73]. SIRT1 also deacetylates AP-1, and represses IKK- and JNK-related pathways, which further contributes to its cardiometabolic health promoting properties [74]. In addition, SIRT1 contributes to the amelioration of oxidative stress via modulation of transcriptional activity of PGC-1α, FOXO3a, and NRF2, resulting in the upregulation of antioxidant enzymes [75,76]. The beneficial effects of SIRT1 in the vasculature protect against atherosclerosis. The main mechanisms include: improvement of endothelial function through activation of eNOS, decreased expression of endothelial adhesion molecules and endothelial tissue factor through deacetylation of p65 subunit of NF-kappa B, and decreased oxidative stress through induction of antioxidant enzymes. In addition, SIRT1 promotes deacetylation of p65 subunit of NF-kappa B in macrophages from the sub-endothelium, leading to repression of LOX-1, a scavenging receptor for oxLDL, and prevention of foam cell formation [77]. It is of note that we identified *LOX-1* (*OLR1* is the official gene name) as significantly modulated by polyphenols within both mRNAs extracted from the literature and predicted miRNA targets retrieved with our bioinformatic analyses, which strongly indicates its importance in the atheroprotective effects of polyphenols. Besides resveratrol that has been extensively studied as a SIRT1 activator, other SIRT1 activators and NAD^+^ boosting compounds also demonstrate promising effects on cardiometabolic health [78].

In addition to their ability to modulate the expression of protein-coding genes, polyphenols are also capable of modulating miRNA expression, which has also been demonstrated in our systematic literature search. Namely, we have identified miRNAs that are involved in inflammation associated with cardiometabolic disorders as significantly modulated with polyphenol intervention. For example, it has been demonstrated that grape polyphenols enriched with resveratrol significantly upregulate miR-21 and miR-181b in PBMCs of diabetic men with hypertension and coronary artery disease, which was accompanied with beneficial cardiometabolic outcomes (Table 1). These results are in line with the data from human studies that report decreased miR-21 in PBMCs in obesity, which is inversely correlated with TNFα and IL6 secreted by PBMCs [79], as well as decreased plasma miR-21 in hypertensive subjects [80]. Mechanisms underlying the beneficial effects of miR-21 on cardiometabolic health have been explored in both in vitro and animal models. More specifically, it has been demonstrated that over-expression of miR-21 in insulin-resistant adipocytes significantly increases the insulin-stimulated glucose uptake via modulation of PTEN-AKT pathway [81], whereas its over-expression in livers of diabetic mice suppresses hepatic gluconeogenesis and improves glucose tolerance [82]. The data for miR-21 are in concordance with the data that are available for miR-181b. For example, miR-181b is significantly decreased in livers and plasma of diabetic mice [83], and in aortas of older mice [84], whereas in patients with poorly controlled type 2 diabetes, plasma miR-181b negatively correlates with the indicators of pro-coagulant and pro-inflammatory state [85].

Opposite to miR-21 and miR-181b, the upregulation of miR-34a is associated with impaired cardiometabolic health. For example, it has been reported that circulating miR-34a is increased in hypertensive subjects [80], as well as in obese children with non-alcoholic fatty liver disease (NAFLD) and/or insulin resistance [86]. Moreover, miR-34a is upregulated in PBMCs from patients with type 2 diabetes [87], and in atherosclerotic plaques in humans and apoE deficient mice [88]. In atherosclerosis, a central role has been ascribed to macrophage miR-34a, as a key regulator of macrophage cholesterol homeostasis and inflammation [88]. Some of the mechanisms underlying the detrimental effects of miR-34a on cardiometabolic health have also been demonstrated in inflamed and dysfunctional adipose tissue in obesity. Indeed, in addition to the progressive increase of miR-34a in mouse epididymal white adipose tissue upon administration of high-fat diet, it has also been demonstrated that in obesity the hypertrophic adipocytes’ exosomal miR-34a suppresses the IL4-induced polarization of macrophages into anti-inflammatory M2 phenotype, by targeting the transcription factor KLF4 [89]. Importantly, KLF4 is among the transcription factors that are identified with our bioinformatic analysis as significantly associated with polyphenol modulated genes (Figure 5A). Accordingly, there is experimental evidence that pre-treatment with olive oil polyphenol hydroxytyrosol prevents TNFα-induced upregulation of miR-34a in cultured human adipocytes and adipocyte-derived exosomes, with concomitant prevention of inflammation and oxidative stress [90].

Bioinformatic analyses demonstrated that identified miRNA targets play a role in different cellular functions and cell signaling. Among the pathways identified are those regulating actin cytoskeleton, focal adhesion, adherens, and gap junction, actin organization and cell adhesions. The adhesion of immune cells to vascular endothelium and their trans-endothelial migration are controlled by the combined action of these pathways. The permeability-regulating factors act through small GTPases that regulate the architecture of the cytoskeleton, which impacts the morphology of the cell and cell–cell junctions and facilitates cell transmigration [91]. Proatherogenic stimuli such as diabetes, dyslipidemia, and oxidative stress initiate impairment of endothelial function resulting in vascular dysfunction that leads to development of atherosclerotic disease, the initial step for multiple cardiovascular disorders. Proinflammatory stimuli cause significant disruption of the endothelial barrier and increased junctional permeability which facilitates trans-endothelial migration of immune cells to the arterial intima and induction of vascular inflammation [92]. Our analysis suggests that polyphenols, by regulating expression of miRNAs targeting genes in these pathways, can prevent or diminish transmigration of immune cells and consequently the development of vascular dysfunction. This observation can be corroborated with a few studies which showed that exposure of endothelial cells to flavanol metabolites decreases adhesion and transmigration of immune cells [67,68]. Moreover, several pathways related to neuronal function have been identified, such as axon guidance or glutamatergic synapse. It has been shown that genes that are expressed in neuronal cells could also be expressed in other type of cells. For example, *PPP2R3A*, expressed also in blood cells, was found to promote activation of NF-kappa B via coupling to Gα12/13, the small GTPase protein RhoA and RhoA-activated kinase, mechanism which increases expression of adhesion molecules and inflammatory mediators [93]. Other genes, such as *GLUL*, *SLC1A2*, *HOMER1*, or *GRIN2D* were observed to be expressed in immune cells (from GEO database (https://www.ncbi.nlm.nih.gov/geoprofiles, accessed on 5 November 2020)). Expression of some of these genes has been observed to be affected in patients with neurological disorders. For example, *HOMER1* gene was observed to be expressed in patients with Alzheimer’s diseases (https://www.ncbi.nlm.nih.gov/geoprofiles/35623805, accessed on 5 November 2020), suggesting that polyphenols could also affect neurological disorders. Indeed, it has been suggested that polyphenol consumption could be involved in prevention of neurodegenerative disorders and improvement of cognitive function [94,95].

## 5. Conclusions

In summary, this systematic literature search and the subsequent integrative bioinformatic analyses allowed us to add new value to the existing data. Applied bioinformatic methods transformed the list of polyphenols modulated genes into valuable sources of information for a better understanding of some of the molecular mechanisms of action of polyphenols, but also toward the identification of potential target genes for future nutrigenetic studies.

## Figures and Tables

**Table 1:** Human intervention studies on nutrigenomic effects of polyphenols, associated with positive cardiometabolic outcomes.

Intervention			Participants				Study Design and Outcomes Related to Cardiometabolic Health		Gene Expression; Significantly Modulated Genes					Ref.
Plant Food/Extract/Bioactive	Dose	Duration of Intervention	Gender	Age (Years)	Number of Volunteers	Health Status	Study Design	Significantly Altered Biomarkers	RNA Type Studied	Method	Cells Analyzed for Gene Expression	Official Gene Symbol	Official Gene Name (from GeneCards)	
Olive oil polyphenols	25 mL olive oil/day with high polyphenol content (366 mg/kg) vs. 25 mL olive oil/day with low polyphenol content (2.7 mg/kg)	3 weeks	M	20–60	18	Healthy	Randomized, crossover, controlled study	Decreased diastolic blood pressure, BMI, total cholesterol, LDL-c, oxLDL, MCP1	mRNA	RT-qPCR	PBMCs	*CD40LG*	CD40 Ligand	[51]
												*IL23A*	Interleukin 23 Subunit Alpha	
												*IL7R*	Interleukin 7 Receptor	
												*CXCR1*	C-X-C Motif Chemokine Receptor 1	
												*ADRB2*	Adrenoceptor Beta 2	
												*OLR1*	Oxidized Low Density Lipoprotein Receptor 1	
Olive oil polyphenols	30 mL olive oil with high polyphenol content—HPC (961 mg/kg) vs. 30 mL olive oil with moderate polyphenol content—MPC (289 mg/kg)	5 h acute study	F, M	20–75	13	Prehypertension or stage 1 hypertension without antihypertensive treatment	Randomized, double-blind, crossover, controlled study	Decreased glucose and oxidized LDL after both interventions. Multiple regression analyses showed that with HPC intervention changes in gene expression were related to a decrease in oxidized low-density lipoproteins and with an increase in oxygen radical absorbance capacity and olive oil polyphenols. These associations were not found after MPC ingestion.	mRNA	RT-qPCR	White blood cells	*ABCA1*	ATP Binding Cassette Subfamily A Member 1	[52]
												*SCARB1*	Scavenger Receptor Class B Member 1	
												*MED1*	Mediator Complex Subunit 1	
												*PPARA*	Peroxisome Proliferator Activated Receptor Alpha	
												*PPARG*	Peroxisome Proliferator Activated Receptor Gamma	
												*PPARD*	Peroxisome Proliferator Activated Receptor Delta	
												*CD36*	CD36 Molecule	
												*PTGS1*	Prostaglandin-Endoperoxide Synthase 1	
Olive oil polyphenols	25 mL olive oil/day with high polyphenol content (366 mg/kg) vs. 25 mL olive oil/day with low polyphenol content (2.7 mg/kg)	3 weeks	M	20–60	18	Healthy	Randomized, double-blind, crossover, controlled study	Decreased diastolic blood pressure, total cholesterol, LDL-c and oxLDL	mRNA	RT-qPCR	PBMCs	*CXCR2*	C-X-C Motif Chemokine Receptor 2	[53]
Resveratrol	800 mg/day	2 months	F, M	30–70	46	Type 2 diabetes	Randomized, double-blind, placebo-controlled, parallel study	Increased plasma total thiol and total antioxidant capacity. Decreased plasma protein carbonyl, systolic and diastolic blood pressure, body weight, BMI, and intracellular superoxide anion in PBMCs.	mRNA	RT-qPCR	PBMCs	*NFE2L2*	Nuclear Factor, Erythroid 2 Like 2	[54]
												*SOD2*	Superoxide Dismutase 2	
Quercetin	1000 mg/day	12 weeks	F	20–40	78	Overweight or obese with polycystic ovary syndrome	Randomized, double-blind, placebo-controlled, parallel study	Decreased plasma resistin	mRNA	RT-qPCR	PBMCs	*RETN*	Resistin	[55]
Curcumin	5 g	2 h acute study	F, M	50–64	5, 5	Healthy smokers, postmenopausal	Randomized, double-blind, placebo-controlled, crossover study	Increased FMD, decreased pulse pressure	mRNA	RT-qPCR	PBMCs	*CXCR6*	C-X-C Motif Chemokine Receptor 6	[56]
												*CXCR3*	C-X-C Motif Chemokine Receptor 3	
												*CXCL9*	C-X-C Motif Chemokine Ligand 9	
												*CXCL17*	C-X-C Motif Chemokine Ligand 17	
												*CXCL16*	C-X-C Motif Chemokine Ligand 16	
												*CXCL10*	C-X-C Motif Chemokine Ligand 10	
												*CX3CR1*	C-X3-C Motif Chemokine Receptor 1	
												*CCR7*	C-C Motif Chemokine Receptor 7	
												*CCR1*	C-C Motif Chemokine Receptor 1	
												*CCL3*	C-C Motif Chemokine Ligand 3	
												*RAC1*	Rac Family Small GTPase 1	
												*PLA2G7*	Phospholipase A2 Group VII	
												*PECAM1*	Platelet And Endothelial Cell Adhesion Molecule 1	
												*PCDH12*	Protocadherin 12	
												*ITGB3*	Integrin Subunit Beta 3	
												*ITGB2*	Integrin Subunit Beta 2	
												*ITGA5*	Integrin Subunit Alpha 5	
												*ICAM3*	Intercellular Adhesion Molecule 3	
												*ICAM2*	Intercellular Adhesion Molecule 2	
												*GJA3*	Gap Junction Protein Alpha 3	
												*CD40*	CD40 Molecule	
												*ABCG1*	ATP Binding Cassette Subfamily G Member 1	
												*ABCC2*	ATP Binding Cassette Subfamily C Member 2	
												*ABCB4*	ATP Binding Cassette Subfamily B Member 4	
												*ABCA4*	ATP Binding Cassette Subfamily A Member 4	
												*ABCA2*	ATP Binding Cassette Subfamily A Member 2	
												*ADIPOR1*	Adiponectin Receptor 1	
												*ADIPOR2*	Adiponectin Receptor 2	
												*FASN*	Fatty Acid Synthase	
												*LIPA*	Lipase A, Lysosomal Acid Type	
												*IL6*	Interleukin 6	
												*STAT1*	Signal Transducer And Activator Of Transcription 1	
												*CCL20*	C-C Motif Chemokine Ligand 20	
												*CCL22*	C-C Motif Chemokine Ligand 22	
												*CCR5*	C-C Motif Chemokine Receptor 5 (Gene/Pseudogene)	
												*CXCL6*	C-X-C Motif Chemokine Ligand 6	
												*ABCA2*	ATP Binding Cassette Subfamily A Member 2	
												*IL1R2*	Interleukin 1 Receptor Type 2	
Grape extract (GE) or grape extract plus resveratrol (GE-Res)	1 capsule/day of GE, GE-Res or placebo in the morning for the first 6 months, and 2 capsules/day for the following 6 months. The phenolic content of the GE and the GE-Res was very similar (151 ± 17 mg and 139 ± 18 mg phenolics per capsule, respectively) but GE-Res also contained 8.1 ± 0.5 mg of resveratrol per capsule.	1 year	M	Adults, up to 80 years old	18	Type 2 diabetes, hypertension, and coronary artery disease	Randomized, triple-blind, placebo-controlled, dose-response, 1-year follow-up study with three parallel arms designated as placebo (maltodextrin), GE (conventional grape extract) and GE-Res (grape extract containing resveratrol)	The following data is extracted from the previous paper [58]: 1. GE-Res vs. Placebo-increased adiponectin, decreased PAI1, total cholesterol, glucose and HbA1c	mRNA, miRNA	RT-qPCR	PBMCs	*IL1B*	Interleukin 1 Beta	[57]
												*TNF*	Tumor Necrosis Factor	
												*NFKBIA*	NFKB Inhibitor Alpha	
												hsa-miR-21-5p		
												hsa-miR-181b-5p		
												hsa-miR-663a		
												hsa-miR-30c-2-3p		
												hsa-miR-34a-5p		

## Data Availability

Not applicable.

## Supplementary Materials

The following are available online at https://www.mdpi.com/article/10.3390/nu13072326/s1, Table S1. Integration of mRNAs and miRNA Targets: Genes from both gene sets that belong to the top 20 enriched terms identified using the bioinformatic tool Metascape.
